# Research Progress in the Application of Exosomes in Immunotherapy

**DOI:** 10.3389/fimmu.2022.731516

**Published:** 2022-02-15

**Authors:** Xiaofang Zhang, Donggang Xu, Yingqiu Song, Rong He, Tianlu Wang

**Affiliations:** ^1^Department of Radiotherapy, Cancer Hospital of China Medical University, Liaoning Cancer Hospital and Institute, Shenyang, China; ^2^Second Clinical Department, Shengjing Hospital, China Medical University, Shenyang, China

**Keywords:** exosomes, immunomodulatory, immunotherapy, wireless communication, molecules transmitting

## Abstract

Exosomes are nanoscale extracellular vesicles (EVs), which are present in all body fluids tested. They are secreted by a variety of cells including macrophages, dendritic cells, mast cells, granulocytes, lymphocytes, and tumor cells. Exosomes secreted by different cells have different biological components and functional characteristics and play an important role in many pathophysiological activities. Recent studies have revealed that exosomes can regulate the occurrence and development of inflammatory immune diseases and tumors by transmitting their unique proteins, lipids, and nucleic acids as signaling molecules to other cells. Exosomes serve as a novel class of diagnostic biomarkers and drug delivery systems with promising applications in immunotherapy, particularly because breakthroughs in nanotechnology have led to the development and exploration of engineered exosomes for immunotargeted therapies. Therefore, here we review the progress being made on the application of exosomes in immunotherapy and its multiple regulatory mechanisms and explore the potential application of exosomes in immunotherapy in the future.

## Introduction

The concept of exosomes was first proposed in 1987, when Johnstone RM and others defined the vesicle structure released by reticulocytes *in vitro* as exosomes (1). Exosomes are extracellular vesicles (EVs) with a diameter of ~40 to 160 nm (average ~100 nm) and an endosomal origin. Exosomes are associated with immune responses, viral pathogenicity, pregnancy, cardiovascular diseases, central nervous system-related diseases, and cancer progression (2). Today, researchers can access exosome research data from databases such as Exocarta (www.exocarta.org) or Vesiclepedia (www.microvesicles.org). Exosome sources are very extensive and include various types of cells, such as intestinal epithelial, apoptotic, mast, endothelial, red blood, and mesenchymal stem cells, macrophages, dendritic cells, lymphocytes, umbilical cord blood stem cells, placental trophoblast and adipose stem cells, and astrocytes (3–16). They can also be derived from various body fluids, such as serum, plasma, sweat, urine, breast milk, semen, and bronchoalveolar lavage fluid (17–23), and parasites, such as polymorphic spiral nematodes and hookworms (24, 25). Different sources of exosomes and their constituents play differential roles in immune regulation (**Table 1**).

**Table 1:** Exosomal molecules and their role in immune modulation.

Molecule	Cell of origin	Effect	Reference
**wt-p53, miR-125b**	Lung adenocarcinoma cell	mediate repolarization of macrophages to an antitumor M1 phenotype	(26)
**M1NVs**	M1 macrophages	repolarize M2 TAMs to M1 macrophages and induce antitumor immune responses	(7)
**miR-125b-5p**	Melanoma	mediates macrophages towards a tumor-promoting TAM phenotype	(27)
**miR-23a-3p**	HCC	upregulates PD-L1 expression and further inhibit T cell function	(28)
**miR-155, miR-146a**	DC	reprogram the cellular response to endotoxin	(29)
**miR-155**	CT26 cell	induces immunogenic DCs, activates and proliferates lymphocytes as well as LPS	(30)
**miR574**	plasma	activates pDC maturation, produces IFN-α and proinflammatory cytokines	(31)
**_**	bone marrow mesenchymal stromal cells	induces immature and mature DCs into a tolerogenic DC population	(32)
**TNF-α, IFN-γ, CCL5, CXCL9, CXCL10, IL-2, IL-6, IL-10, IL-12, IL-15, IL-17**	serum	induce differentiation of Th1/Th2 cells, enhance proliferation and migration of T lymphocytes	(19)
**galectin-1 protein**	head and neck cancer	induces a suppressor phenotype in CD8+T cells	(33)
**Classical MAPK proteins include p38, ERK, and JNK**	Pancreatic cancer	induce ER stress-mediated apoptosis of T lymphocytes	(34)
**let-7d**	Treg cell	inhibits Th1 cell proliferation and cytokine secretion	(35)
**TPO, HSP60, MHC-II**	thyroid cell	Promote CD4+ T lymphocyte expression and release of pro-inflammatory cytokines	(36)
**IL-35**	i35-Bregs cell	inhibits the expansion of pathogenic Th17 cells	(37)
**miR-142-3p, miR-142-5p, miR-155**	T cell	promote lymphocyte recruitment and increase islet β cell death	(38)
**miR-490**	mast cell	inhibits metastasis and invasion of HCC cells	(39)
**TGFβ-1**	mast cell	affects the migratory phenotype of induced bone marrow mesenchymal stem cells	(40)
**miR-409-3p**	mast cell	promotes microglia migration, activation, and neuroinflammation	(41)
**_**	eosinophil	affects the function of structural lung cells and contributes to the remodeling process of asthma	(42)
**miR-29a, miR-92a**	glioma	induce the expansion of bone marrow-derived suppressor cells	(43)
**miR-375**	glioma	inhibits proliferation, migration, and invasion of gliomas	(44)
**gp130**	breast cancer	induces IL-6 secretion and prosurvival phenotype of macrophages	(45)
**miR-9, miR-181a**	breast cancer	promote the development of early-stage MDSCs	(46)
**miR-378a-3p, miR-378d**	breast cancer	promote breast cancer stemness and chemoresistance	(47)
**miR-501-3p**	pancreatic ductal adenocarcinoma	promotes progression of pancreatic ductal adenocarcinoma	(48)
**miR-1468-5p**	cervical cancer	promotes lymphangiogenesis and evasion of anticancer immunity	(49)
**miR-6089**	rheumatoid arthritis	modulates LPS/TLR4-mediated inflammatory responses	(50)
**miR-424**	rheumatoid arthritis	Increases the number of Th17 cells and decreases the number of Treg cells	(51)
**miR-223**	mast cell	disrupts intestinal barrier function by inhibiting CLDN8 expression	(52)
**myelin oligodendrocyte glycoprotein (MOG)**	serum	enhance the antimyelin immune response in multiple sclerosis	(53)
**_**	bone marrow mesenchymal stromal cells	suppress symptoms of inflammation and demyelination in the central nervous system	(54)
**leukotriene synthase**	Macrophages, DC	regulates the biosynthesis of LTs and the recruitment of granulocytes	(55)
**_**	lung epithelial cells	promotes the expansion and chemotaxis of undifferentiated macrophages	(56)
**_**	bone marrow mesenchymal stromal cells	promotes proliferation and immune-modulating activity of Tregs	(57)
**TGF-β2**	fibroblasts	promotes airway remodeling	(58)
**miR-338-5p**	serum	inhibits IL-6 mRNA levels and inflammation	(59)
**miR-1260b**	gingival tissue-derived MSCs	induce anti-inflammatory M2 macrophage polarization	(60)

Initially thought to be a means of disposing waste products produced during cell maturation (61), exosomes are now thought to regulate inflammatory responses by modulating cytokines secreted by immune cells. With access to the interstitial space and ultimately to the circulation, exosomes represent an extensive signaling system in crosstalk between cells and organs. Secreted exosomes from specific cells are able to act on receptor cells at distal sites, displaying many of the features of classical endocrine signaling (62). They have the capacity for signal transduction and can initiate communication between cells. Simultaneously, exosome miRNAs play an important role in regulating tumorigenesis and metastasis. In addition, exosomes are emerging biomarkers and novel targeted drug delivery systems and have potential clinical applications in the diagnosis and treatment of many diseases (63).

Recent retrospective studies on exosomes have mostly focused on the therapeutic aspects of cancer and inflammation and at the molecular level of exosomes, mainly on cytokines and RNA. Both immune and non-immune cells are capable of secreting exosomes. Pegtel et al. (64) discovered new insights in the analysis of the structural components of exosomes and mechanisms by which they are generated, suggesting that the existence of an endosomal model of exosome biogenesis does not imply that all exosomes can only be produced through endosomal outgrowth. Furthermore, recent advances have shown that exosome-mediated intercellular communication of extracellular RNA affects the behavior of individual immune cells and is involved in local and systemic immune responses (65). Nation et al. (66) highlighted a series of exosome-dependent signaling pathways that could serve as targets for cancer-targeted therapies. In addition, previous research reviews have typically explored the immunomodulatory effects of exosomes of the same immune or tumor cell origin on cancer or inflammation and have tended to be limited to mechanisms and therapeutic strategies in cancer (67–69). With the study of the multiple roles of exosomes in the development of neoplastic diseases, it has become evident that exosomes have both promotive and inhibitory effects on cancer (70). Here, we provide a more comprehensive and organized summary of the different mechanisms of immune action of exosomes from different sources on the same immune cells or on the same disease including major oncological, autoimmune, and inflammatory diseases and their application in immunotherapy.

## Exosomal Regulation of Organism Immunity

### Immunomodulatory Effects of Exosomes on Macrophages

Tumor-associated macrophages (TAMs) are the most abundant immune cells infiltrating tumor tissue and can contribute to their production, development, metastasis, and immune escape through a variety of pathways. However, TAMs usually have two contrasting phenotypes: the antitumor M1 and protumor M2 subtypes (71). Under different pathological conditions, macrophages can exhibit heterogeneity across successive polarization states, that is, they have the ability to repolarize from one phenotype to another (72), and exosome-mediated repolarization of macrophages may serve as a novel therapeutic technique for a variety of diseases. For example, Trivedi et al. (26) showed that exosomes (i.e., wt-p53/exosome, miR-125b/exosome, and combination/exosome) have a reprogrammed miRNA profile in which miRNAs are primarily associated with the triggering of apoptosis-related genes and p53 signaling and can promote repolarization of macrophages to the antitumor M1 phenotype. Exosomes derived from M1 macrophages (M1NVs) can repolarize M2 TAMs to M1 macrophages, releasing proinflammatory cytokines and inducing antitumor immune responses (7). Recently, Gerloff et al. (27) showed that miR-125b-5p secreted by cutaneous melanoma-derived exosomes induce macrophages to develop a tumor-promoting TAMs phenotype by targeting the LIPA 3’UTR. Furthermore, influencing exosome release by modulating the tumor microenvironment can regulate the function of immune cells such as macrophages, thereby promoting tumor survival, progression, and metastasis. Liu, J et al. (28) reported that endoplasmic reticulum stress led to the release of exosomal miR-23a-3p from hepatocellular carcinoma cells and upregulated PD-L1 expression in macrophages, which in turn suppressed T-cell function *via* the exosomal miR-23a-PTEN-AKT pathway. Although the above studies suggest that exosomes have the potential to mediate the repolarization of macrophages or influence their immune function and, thus, modulate antitumor immunity, more studies are needed to fully elucidate the immunomodulatory effects of exosomes on TAMs.

### Immunomodulatory Effects of Exosomes on Dendritic Cells

Dendritic cells (DCs) are the most functional antigen precursor cells, and the release of exosomes with dissimilar miRNAs by DCs depends on the degree of their maturation (73). Immature DCs downregulate T-cell reactions to effect maintenance of immune resistance, while mature DCs enhance immunity. To enhance their functions, DCs communicate with neighboring DCs through intercellular contacts, vesicle exchange, and soluble medium. Alexander et al. (29) showed that endogenous miR-155 and miR-146a are released from the exosomes of DCs and subsequently taken up by recipient DCs. After ingestion, exogenous microRNAs induce the repression of target genes and can reprogram the cellular response to endotoxin, where exosomal delivery of miR-155 upregulates while miR-146a downregulates the expression of inflammatory genes. Exosomes function in crosstalk between DCs and likewise link communication between other cells and DCs. For example, tumor cell-derived exosomes can act as carriers to deliver exogenous miRNA-155 into DCs, thus enabling simultaneous antigen triggering and delivery of miRNAs to DCs (30). It has also been demonstrated that within exosomally delivered microRNAs in the plasma of patients with systemic lupus erythematosus (SLE) are potential novel TLR7 endogenous ligands that induce plasma cell-like DC (pDC) activation and the production of IFN-α and proinflammatory cytokines (31). Thus, microRNAs may be novel pathogenic mediators for initiating autoimmune responses and potential therapeutic targets for the treatment of type I interferon-mediated diseases. Recently, Shahir et al. (32) found that endotoxin-treated mesenchymal stem cell (MSC)-derived exosomes induced the differentiation of immature and mature DCs into a tolerogenic DC population, i.e., decreased the expression of DC surface markers and lymphocyte proliferation and IL-6 release and increased IL-10 and TGF-β release. However, low targeting and specificity greatly limit the effectiveness of exosome delivery. Therefore, Li et al. (74) designed an RNA delivery system based on MSC-derived exosomes capable of targeting specific binding of DCs and promoting HLA-G expression, where HLA-G can inhibit DC-triggered allogeneic T cell proliferation, thereby inducing long-term immune tolerance. Thus, exosomes of diverse origins can regulate DC differentiation, maturation, and function and may be important regulators of DC-induced immune responses.

### Immunomodulatory Effects of Exosomes on Lymphocytes

In addition to macrophages and dendritic cells, exosomes play an important role in the biology of lymphocytes, regulating their activation, differentiation, proliferation, chemotaxis, and mediation of diseases. Gao et al. (19) showed that cytokine/chemokine-rich exosomes are immunoreactive during sepsis and induce Th1/Th2 differentiation, significant T cell proliferation, and migration capacity, which cause protective effects in cecum ligation and puncture mice. In addition, Maybruck et al. (33) found that tumor-derived exosomes induce a suppressor phenotype (SP) in CD8+ T cells, which may act synergistically with exosomal proteins and RNA, making immunosuppressive exosomes a potential therapeutic target for preventing T cell dysfunction and enhancing antitumor immune response. However, the mechanisms by which signal transduction pathway activity in lymphocytes is influenced by exosomes of tumor origin remain unknown. Recently, it has been shown that pancreatic cancer (PC) cell-derived exosomes are taken up by T lymphocytes and activate p38MAPK, which then induces endoplasmic reticulum stress-mediated apoptosis, ultimately leading to immunosuppression (34). Of interest is that exosomal miRNA transfer is a mode of communication between lymphocytes and other immune cells. Okoye et al. (35) demonstrated that non-cell-autonomous gene silencing mediated by miRNA-containing exosomes is the mechanism by which Treg cells are able to inhibit T cell-mediated disease development. miRNAs are transferred by Treg cells to Th1 cells, thereby inhibiting their multiplication and the production of cytokines. In addition, influencing lymphocyte expression and proliferation directly or indirectly through exosomes affects the development of some autoimmune diseases. For example, Cui et al. (36) found that IFN-γ-treated thyroid cell-derived exosomes containing TPO, HSP60, and MHC-II *in vitro* targeted the activation of dendritic cells, which in turn promoted the expression and release of proinflammatory cytokines IFN-γ, IL-17A, and IL-22 by CD4+ T lymphocytes and inhibited the expression and release of anti-inflammatory cytokines IL-4, IL-10, and TGF-β1, ultimately suppressing thyroiditis (36). Kang et al. (37) demonstrated that i35-Bregs cells release IL-35-containing exosomes, which aid in suppressing severe uveitis by inhibiting the expansion and transport of pathogenic Th17 cells to the retina. On the other hand, Guay et al. (38) found that T cells can release exosomes containing miR-142-3p, miR142-5p, and miR-155 and transfer their inactive form to pancreatic β-cells, specifically triggering the expression of their apoptosis- and chemokine signaling-related genes, promoting lymphocyte recruitment and exacerbating β-cell death, thereby inducing the development of type 1 diabetes.

### Immunomodulatory Effects of Exosomes on Mast Cells and Eosinophils

Mast cells (MC) are multifunctional master cells implicated in the innate and adaptive immune response, and their role is best exemplified in allergic and anaphylactic responses. MCs are both degranulated and restored and are a rich source of exosomes that have attracted much attention in recent years. The upregulation of miR-490 expression levels in MC-derived exosomes stimulated by HCV-E2 and transferred to hepatocellular carcinoma (HCC) cells inhibit tumor metastasis by reducing the activity of the EGFR/AKT/ERK1/2 pathway, thereby suppressing invasion of HCC cells (39). It has also been shown that mast cells have antiproliferative activity against melanoma cells, which is dependent on trypsin-like proteases (a tetrameric protease stored in MC granules). Melo et al. (75) found that trypsin-like proteases were phagocytosed by melanoma cells, and DNA-encapsulated exosomes were released, which were then translocated to the nucleus, inducing the execution of histone 3 shearing, degradation of Lamin B1 and hnRNP A2/B1, and extensive nuclear remodeling, resulting in the downregulation of expression of the oncogene EGR1 and a variety of non-coding RNAs, including oncogenic species. Furthermore, Shelke et al. (40) showed that MC-derived exosomes are modified by latent TGFβ-1 on their surface and retained in recipient bone marrow MSC endosomes, inducing a migratory phenotype of recipient cells by triggering a SMAD2-dependent pathway. It is evident that MC exosomes can transmit signals in endosomes by delivering bioactive surface ligands to intracellular compartments. In addition, the exosomal miR-409-3p secreted by activated MCs promotes microglia relocation and activation and neuroinflammation by targeting Nr4a2 to stimulate the NF-κB pathway (41), suggesting that exosomes and cytokines are involved in the interaction between MCs and microglia to cause neuroinflammation.

Eosinophils represent a small number of circulating granulocytes that mainly infiltrate helminth infections or metaplastic inflammation, are central to the pathogenesis of asthma, and release a variety of (pre-formed) effector molecules mainly attributed to physiological and pathogenic effects. In 2015, Mazzeo et al. (76) used transmission electron microscopy (TEM) to demonstrate the presence of multivesicular bodies in eosinophils. It was further found that eosinophils secrete exosomes in patients with asthma and that the release of exosomes to extracellular mediators increases after IFN-γ stimulation. These findings suggested that exosomes may play an essential role in the development of asthma, causing them to be considered as biomarkers. Later, Cañas et al. (25) found that asthmatic eosinophil-derived exosomes induced increased apoptosis of tracheal epithelial cells at 24 and 48 h, impeding trauma closure. After 72 h, increased proliferation of bronchial smooth muscle cells was associated with increased levels of ERK1/2 phosphorylation (42). Thus, eosinophil-derived exocytosis in patients with asthma is actively involved in the development of asthma pathological features through structural lung cells. However, the exact role of eosinophil-derived exosomes in the biological processes leading to asthma has not been fully determined.

## Immunotherapeutic Role of Exosomes

All cells in an organism secrete EVs, and exosomes are nanoscale EVs that can play a significant regulatory role in various pathological processes (e.g., immune responses and tumorigenesis). For example, metastatic melanoma releases high levels of EVs, mainly in the form of exosomes, which carry PD-L1 on their surface. Exosomal PD-L1 can bind directly and inhibit T-cell function like a drone (77). At the same time, exosomes are composed of natural lipid bilayers and rich in adhesion proteins, which can easily interact with cell membranes and are well suited as carriers for drug transport. Therefore, the potential clinical applications of exosomes as biomarkers for disease diagnosis and drug delivery carriers for therapy have attracted much attention in the past few years. For example, exosomal miRNAs are emerging as key regulators of the development of diabetes mellitus (DM) and can regulate the expression levels of related genes, mainly involved in pancreatic β-cell damage and insulin resistance, implying that it may be possible to treat DM by silencing or activating exosomal miRNAs in patients with DM in an exogenous manner (78).

### Exosomes and Treatment of Neoplastic Diseases

#### Exosomes and Glioma

Glioma is a heterogeneous, complex brain tumor often with a poor prognosis, originating in the central nervous system. The clinical management of malignant gliomas remains a major challenge owing to the highly infiltrative growth and chemoresistance of the tumor and the presence of the blood-brain barrier (BBB) (79). The ability of exosomes to cross the BBB and be easily produced in almost all types of human biological fluids make them potential biomarkers for gliomas. Exosomal non-coding RNAs (ncRNAs) in gliomas include microRNAs, lncRNAs, and circRNAs, which can be selectively encapsulated, released, and delivered in exosomal cells to regulate many features of gliomas, such as multiplication, infiltration, vascular growth, immune evasion, and therapeutic tolerance (80). Myeloid-derived suppressor cells (MDSCs) play a key role in mediating the formation of an immunosuppressive environment and assisting tumors to evade host immune responses. However, Guo et al. (43) reported that glioma-derived exosomes (GEXs) can stimulate the differentiation of functional MDSCs by transferring miR-29a and miR-92a to MDSCs both *in vitro* and *in vivo*, and that hypoxia-induced GEXs have a stronger ability to induce MDSCs than normoxia-induced GEXs. However, the question of whether miR-29a and miR-92 can be essential biomarkers useful in the diagnosis and prognosis of glioma remains unanswered. Xu et al. found that the removal of the oncogene miR-375 from glioma cells by exosomes leads to the continuous activation of the CTGF-EGFR oncogenic pathway, which promoted the multiplication, metastasis, and aggression of glioma (44), indicating that exosomal miR-375 may be a promising glioma biomarker. Furthermore, taking advantage of the inherent inflammatory chemotaxis of neutrophils and their excellent BBB crossing ability, Wang et al. (81) proposed a neutrophil-exosome (NEs-Exos) drug delivery system containing adriamycin (DOX), which could rapidly permeate the BBB and metastasis into the brain to target aggressive tumor cells in inflammatory brain tumors, effectively suppressing tumor growth and prolonging survival. PD-L1 on exosomes may be another mechanism by which glioblastoma suppresses antitumor immunity. Ricklefs et al. (82) first identified glioblastoma exosomes containing PD-L1 and blocking TCR-induced T-cell activation. Glioblastoma can upregulate PD-L1 expression upon stimulation with IFNγ, which in turn increases PD-L1 levels on exosomes, thereby inhibiting T-cell activation.

#### Exosomes and Breast Cancer

In recent years, there have been several research advances in the application of exosomal immunotherapy in breast cancer. Ham et al. (45) showed that breast cancer-derived exosomal gp130 activates the IL-6/STAT3 pathway in macrophages and causes the expression of protumor cytokines, thus potentially tilting macrophages toward a procancer phenotype, suggesting that breast cancer-derived exosomal proteins may play a key role in cancer progression. Moreover, Nabet et al. (83), found that triggering stromal NOTCH-MYC in breast cancer cells led to an increase in POL3-driven RN7SL1 expression, which resulted in the production of RN7SL1 in stromal exosomes that were not shielded by the RNA-binding protein SRP9/14. Upon metastasis to breast cancer cells, activation of PRR RIG-I by unscreened RN7SL1 could enhance tumor growth, metastasis, and treatment resistance. Recently, it was found that tumor exosome-derived miR-9 and miR-181a promote the formation of IL-6-high-expressing early myeloid-derived suppressor cells in breast cancer by suppressing key regulators, including SOCS3 and PIAS3, in the negative feedback circuit of the JAK/STAT signaling pathway (46). This may provide a potential therapeutic target for breast cancers with high IL-6 expression. Yang et al. (47) also found that chemotherapy activated the EZH2/STAT3 axis in breast cancer cells, which in turn secreted miR-378a-3p- and miR-378d-rich exosomes that were taken up by surviving breast cancer cells and then activated the WNT and NOTCH stem cell pathways by targeting DKK3 and NAMB, ultimately leading to drug resistance (47). Therefore, blocking the secretion of such exosomes during chemotherapy may reduce the occurrence of drug resistance and, thus, maximize the therapeutic effect of breast cancer therapies.

#### Exosomes and Other Neoplastic Diseases

Ludwig et al. (23) showed that plasma exosomes from patients with head and neck cancer (HNC) carry immunosuppressive molecules that interfere with immune cell function and correlate with disease activity in HNC. They also showed that the presence, number, and molecular content of isolated plasma-derived exosomes could be used to distinguish between patients with HNC and patients with active disease (AD) and those with no apparent disease (NED) after tumor treatment, showing that plasma exosomes can be potential biomarkers of HNC progression (23). Ghosh et al. (84) used Nanosight/TEM/immunoblotting and anti-Alix/anti-GRP78/anti-Asgr2 to validate the presence of HBV/HCV-infected low methemoglobin non-hepatocellular carcinoma (non-HCC) exosomes. They found that in exosomes with miR-21-5p and miR-10b-5p/miR-221-3p/miR-223-3p expression was significantly higher than that of non-HCC patients (84); thus, exosome-encapsulated microRNAs could be used as circulating diagnostic markers for low methemoglobin HCC. Yin et al. (48) demonstrated that in pancreatic ductal adenocarcinoma tissue, M2 macrophage-derived exosomal miR-501-3p inhibited the expression of TGFBR3, a tumor suppressor gene, by activating the transforming growth factor-β signaling pathway, thereby promoting tumor development (48), and possibly providing a new target for the molecular therapy of pancreatic ductal adenocarcinoma. Zhou et al. (49) found that miR-1468-5p encapsulated in exosomes secreted from cervical cancer promoted lymph angiogenesis, upregulated PD-L1 expression, impaired T-cell immunity, directly targeted the SOCS1 promoter in HMBOX1, activated the JAK2/STAT3 pathway in tumor-associated lymphatic endothelial cells (LECs), and enabled cancer cells to evade anticancer immunity. This suggests that exosomal miR-1468-5p can induce an intact immunosuppressive tumor microenvironment in LECs and may be a prognostic biomarker and therapeutic target for cervical cancer.

#### Engineered Exosomes and Neoplastic Diseases

Based on the role of exosomes in disease suppression, Rayamajhi et al. (85) designed hybrid exosomes (HE) by hybridizing exosomes from mouse macrophages with synthetic liposomes. With higher colloidal stability, drug loading, and pH-sensitive drug release, they had different targeting and cytotoxicity capacities to normal and cancer cells and could mimic immune cells to target tumors (85), suggesting that HEs could be a powerful platform for tumor-targeting drug delivery. Additionally, Shi et al. (86) developed genetically engineered exosomes with anti-CD3 and anti-HER2 antibodies to produce SMART- Exos, which showed highly effective and selective induction of tumor-specific immunity expressing HER2 both *in vitro* and *in vivo*, thus creating an innovative class of immunotherapy against HER2-positive breast cancer (86). Interestingly, tumor vaccines are emerging immunotherapies, but their clinical application is hampered by difficulties in neoantigen recognition, immune cell activation, and tumor infiltration. Liu et al. (87) developed a tumor vaccine (hEX@BP) that encapsulates black phosphorus quantum dots with exosomes (HE). In comparison with black phosphorus quantum dots alone, hEX@BP showed better long-term photothermal therapy *in vivo* performance and higher tumor temperature and tumor targeting and promoted the infiltration of T lymphocytes into tumor tissue. In addition, Kim et al. (88) used fusion exosomes to introduce fusion viral antigen (VSV-G) to the surface of xenograft tumor cells, making them more easily recognized and phagocytosed by DCs, thus effectively presenting tumor antigens to T lymphocytes and inhibiting tumor growth (88).

### Exosomes and the Treatment of Autoimmune Diseases

#### Exosomes and Systemic Lupus Erythematosus

SLE is a common type of systemic autoimmune inflammatory connective tissue disease involving almost every organ, mostly occurring in young women, with significant morbidity and mortality rates. It is characterized by the production of multiple antigen-driven autoantibodies against intracellular and extracellular molecules. Lee et al. (89) showed that circulating exosomes from patients with SLE induced a higher production of IFN-α, TNF-α, IL-1β, and IL-6 than exosomes from healthy individuals and that exosome levels correlated with disease activity in SLE patients, whereas depleted SLE serum and mechanically fragmented SLE exosomes both did not induce the production of any significant cytokines (89). Therefore, circulating exosomes of SLE patients have immunological activity and may be used as a new biomarker of SLE disease activity. It is worth mentioning that the overdose of type I interferon is a biomarker for and main pathogenic mechanism of many autoimmune diseases such as SLE. In addition, Salvi et al. (31) reported that microRNA delivered by SLE exosomes activates human plasmacytoid DCs to secrete IFN-α through TLR7, indicating that microRNA can be used as a potential target for the treatment of SLE. Recently, López et al. (90) found that T cell-derived exosomes (CD3-Ex) are related to CD4+CD28- cells. The activation status of the senescent subgroup is related to the interferon score of patients with SLE. Type I interferon signals may promote the excessive activation of leukocytes and inflammatory mediators in patients with SLE (90). In patients with SLE, T cell-derived exosomes are expected to become a promising therapeutic tool for SLE. In addition, Tan et al. (91) showed that the downregulation of serum exosomal miR-451a expression is associated with disease activity and SLE kidney damage related to the role of exosomes in cell-to-cell communication in SLE (91), which also provides a potential strategy for the treatment of SLE.

#### Exosomes and Rheumatoid Arthritis

Rheumatoid arthritis (RA) is a chronic systemic inflammatory disease of unknown etiology. It is an autoimmune disease that mainly affects joints throughout the body, mostly the small joints of the hands, wrists, and feet, resulting in joint deformities of varying degrees, leading to lifelong disabilities and increased mortality. The levels of amyloid A (AA) and lymphatic vessel endothelial cell hyaluronan receptor-1 (LYVE-1) exosomes differed between the clinical remission (CR group) and non-clinical remission groups (non-CR group) in patients with RA, and both serum/exosomal AA and LYVE-1 levels were significantly and positively correlated with C-reactive protein (CRP) levels in the non-CR group (92). Therefore, exosomal AA and LYVE-1 are expected to be effective biomarkers for assessing disease activity in patients with RA. In addition, exosome-encapsulated microRNAs have been recognized as promising biomarkers of autoimmune diseases. Xu et al. (50) found that exosome-encapsulated miR-6089 from patients with RA inhibited lipopolysaccharide (LPS)-induced cell proliferation and activation of macrophage-like THP-1 cells and regulated IL-6, IL-29, and TNF-α production by targeting the TLR4 signaling pathway. It is evident that exosome-encapsulated miR-6089 can regulate LPS/TLR4-mediated inflammatory responses and potentially act as a novel and promising biomarker for RA. Prospective studies of these biomarkers could help develop new diagnostic tools to assess disease activity and prognosis of RA and other autoimmune diseases. Besides, Wang et al. (93) found that miRNAs in exosomes of patients with RA were more abundant than those in exosomes of healthy individuals and that upregulated miR-17 in patients with RA may inhibit Treg induction by suppressing transforming growth factor β receptor II (TGF-βRII) expression. Thus, altered miRNA expression in exosomes of patients with RA may be involved in the pathogenesis of RA by disrupting the homeostasis of Treg cells. It has also been shown that miR-424 expression of exosomes derived from synovial fibroblasts (SFS-EXO) is increased in patients with RA and can significantly induce T cell differentiation by targeting the transcription factor FoxP3, resulting in an increase in the number of Th17 cells and a decrease in the number of Treg cells (51). Therefore, miR-424 may also be a potential therapeutic target for RA. It is worth acknowledging that glucocorticoids (GCs) have a strong therapeutic effect on RA, but the serious adverse effects after long-term administration limit their clinical application. Recently, Yan et al. (94) established a bionic exosome of dexamethasone sodium phosphate nanoparticles (Exo/Dex), resulting in an active targeted drug delivery system that significantly reduced joint inflammation by inhibiting the expression of proinflammatory cytokines and increasing the expression of anti-inflammatory cytokines, hence providing a promising strategy for improving the therapeutic effects of GCs on RA.

#### Exosomes and Inflammatory Bowel Disease

Inflammatory bowel disease (IBD) is a chronic autoimmune disease with intestinal immune dysfunction, primarily including Crohn’s disease (CD) and ulcerative colitis (UC). Eichenberger et al. (24) reported that the interaction of hookworm secreted exosomes with host cells prevented inducible colitis in mice. This suggests that the proteins and miRNAs contained in worm exosomes have great potential applications in the development of drugs for the treatment of worm infections and chronic non-infectious diseases caused by immune system dysregulation, such as inflammatory bowel disease. Additionally, human breast milk is rich in exosomes that may also prevent necrotizing small intestinal colitis (21). Bulek et al. (95) found that epithelial-derived gastrin D-mediated release of IL-1β exosomes was associated with the development of intestinal inflammation. In fact, Lian et al. (96) previously found that “auto-DNA” released by exosomal secretion enters the cytoplasm of innate immune cells and activates AIM2 inflammatory vesicles, leading to the secretion of mature IL-1β and IL-18, intestinal mucositis, and delayed diarrhea. Recent studies have revealed that mast cell-derived exosome-mediated transfer of functional miRNAs from human mast cells-1 (HMCs-1) to intestinal epithelial cells inhibited the expression of tight junction-related proteins (including TJP1, OCLN, CLDN8), thereby significantly increasing intestinal epithelial permeability, disrupting intestinal barrier function, and promoting the development of inflammatory bowel disease (52). These studies provide new insights into the mechanisms of innate immune responses that cause intestinal toxicity and new options for the use of exosomes in the prevention and treatment of disease.

#### Exosomes and Multiple Sclerosis

Multiple sclerosis (MS) is the most common autoimmune disease characterized by inflammatory demyelinating lesions in the white matter of the central nervous system, often resulting in severe physical disability in patients. Selmaj et al. (97) used next-generation sequencing (NGS) technology to analyze the overall RNA profile of serum exosomes comparing patients with relapsing-remitting multiple sclerosis (RRMS) and healthy controls and found that in patients with RRMS, four circulating miRNA classes in serum had significantly lower exosome expression during relapse. The possible targets of these miRNAs include STAT3 as well as the cell cycle regulator aryl hydrocarbon receptor, suggesting that intercellular communication is disrupted in MS (97). This suggests that exosomal miRNAs may be useful biomarkers for differentiating MS relapses. Galazka et al. (53) found that exosomes expressed three major myelin basic proteins, proteolipid proteins, and myelin oligodendrocyte glycoprotein (MOG), which promoted the expansion of MOG-T cell receptor transgenic T cells. This demonstrates that serum exosomes maintain the immunogenicity of MOG and may enhance and/or prolong the antimyelin immune response in MS (53), suggesting that exosomes are involved in the development and progression of MS. Furthermore, exosomes from bone marrow mesenchymal stem cells were found to reduce inflammation and demyelination in the rat central nervous system by modulating the polarization of microglia in an experimental autoimmune encephalomyelitis model (54). Consequently, the application of bone marrow MSC-derived exosomes may be a potential therapeutic approach for the treatment of MS or other inflammatory diseases.

### Exosomes and Treatment of Inflammatory Diseases

#### Exosomes and Asthmatic Bronchitis

Asthma is a complex multifactorial disease influenced by multiple factors and is thought to be a heterogeneous disease characterized by chronic inflammation of the airways involving multiple cells and cellular components. Esser et al. (53) found that exosomes from human macrophages and DCs produce leukotriene biosynthetic enzymes that stimulate granulocyte transfer, suggesting that exosomes can contribute to inflammation by participating in leukotriene biosynthesis and granulocyte recruitment (55). Following this, Paredes et al. (98) found that bronchoalveolar lavage fluid (BALF) exosomes contribute to cytokine and leukotriene production in allergic asthma and may promote subclinical inflammation by increasing cytokine and leukotriene C4 (LTC_4_) production in the airway epithelium of patients with asthma. According to findings by Kulshreshtha et al. (56), under the influence of IL-13, epithelial cell-derived exosomes induced enhanced proliferation and chemotaxis of undifferentiated macrophages in the lungs under inflammatory conditions in asthma. However, GW4869 inhibited the production of exosomes, decreased the proliferation rate of monocytes, and alleviated asthma symptoms. Apparently, airway epithelial cell-derived exosomes can induce the generation and development of asthma inflammation. Du et al. (57) found that exosomes derived from bone marrow mesenchymal stem cells in patients with asthma upregulated the expression of IL-10 and transforming growth factor-β-1 (TGF-β1) in PBMCs, thereby promoting the proliferation and immunomodulation of Tregs (57). Simultaneously, it has been found that the overexpression of transforming growth factor-β-2 (TGF-β2) induces enhanced release of cellular exosomes of TGF-β2, resulting in reduced epithelial cell proliferation. In contrast, exosomes from fibroblasts of patients with severe asthma showing lower TGF-β2 levels contribute to epithelial cell proliferation, thereby promoting airway remodeling (58). This suggests that exosomes play a key role in the immune regulation of bone marrow mesenchymal stem cells and asthma airway remodeling by bronchial fibroblasts, which have the potential for the treatment of asthma.

#### Exosomes and Complex Regional Pain Syndromes

Recent studies suggest a potential pathophysiological mechanism for exosomes in complex regional pain syndrome (CRPS). CRPS is a term describing debilitating pleiotropic pain and dysfunction characterized by chronic inflammation. McDonald et al. (99) showed that miRNAs, altered in the circulation of patients with CRPS, are transported by exosomes. Through their systemic signaling capacity, exosomes can trigger the multifactorial pathological effects of chronic pain. Therapeutic plasma exchange (PE) or plasma replacement is an *in vitro* procedure used to treat immune disorders. Ramanathan et al. (59) identified 17 miRNAs from PE-induced exosomes of patients with CRPS that were significantly different before and after treatment and found that exosomal miR-338-5p levels were higher in PE responders. Overexpression of miR-338-5p can downregulate IL-6 mRNA and protein levels *in vitro*, thereby suppressing inflammation (59). In the same year, they also found, from comparative data of primitive immune cell-derived exosomes from patients with CRPS and controls, that while the relative expression of miR-939 was higher in B, T, and NK cell-derived exosomes than in monocyte-derived exosomes in controls, only B cell-derived exosomes showed significantly higher levels of miR-939 in patients with CRPS (100). These studies suggest that differential miRNA sorting of exosomes and their effects on receptor cell function may contribute to the study of the underlying pathophysiological mechanisms of CRPS. Meanwhile, the strategy of studying exosomal miRNAs to stratify patients with CRPS and, thus, maximize the effectiveness of PE for CRPS has some feasibility.

#### Exosomes and Periodontitis

Periodontitis is a chronic inflammation of periodontal supporting tissues (e.g., gingiva, periodontal membrane, alveolar bone, and dental bone) caused mainly by local factors, such as plaque bacterial infection, which often leads to alveolar bone resorption and progressive tooth loosening. The relationship between periodontitis and circulating exosomal miRNAs is still unclear, but it has been found that the expression levels of serum exosomal miR-207, miR-495, and miR-376b-3p in mice with periodontitis were significantly upregulated after 2 weeks with periodontitis (101). This suggests that these serum exosomal miRNAs could be used as biomarkers for periodontitis. Interestingly, Sundaram (102), found that ginger exosome-like nanoparticles could significantly attenuate the pathogenic mechanism of periodontitis by *Pseudomonas gingivalis* by interacting with heme-binding protein 35 (HBP35) on the surface of *Porphyromonas gingivalis* and being selectively taken up by it (102). This suggests that edible plant exosome nanoparticles can be used as potential therapeutic agents to treat chronic periodontitis. Nakao et al. (60) found that the pretreatment of gingival tissue-derived mesenchymal stem cells (GMSCs) with TNF-α not only increased the number of exosomes secreted by GMSCs, whose exosome miR-1260b could target the WNT5a-mediated NF-κB ligand-receptor activator (RANKL) expression pathway and inhibit its osteolytic activity, but also the expression of CD73 exosomes. This resulted in the induction of anti-inflammatory M2 macrophage polarization (60), which showed that GMSC-derived exosomes pretreated with TNF-α have a significant ability to regulate inflammation and osteoclastogenesis, potentially introducing a new level of treatment for periodontitis. In addition, Wang et al. (103) showed that cyclic retraction force can induce periodontal membrane cells to secrete exosomes, which inhibit LPS-induced NLRP3 inflammasome signaling in human macrophages by suppressing the NF-κB signaling pathway in macrophages. This helps maintain the dynamic balance of periodontal inflammation. Interestingly, P2X7 receptor (P2X7R) gene modification can reverse inflammation-mediated damage to periodontal membrane stem cells. It has recently been found that P2X7R gene-transduced cells can also positively affect other coexisting cells in *in vivo* through an exosome-mediated paracrine mechanism, either by transplanting small amounts of P2X7R gene-modified cells or using their exosomes to act as initiators to alter the local inflammatory microenvironment to aggregate stem cells and promote tissue regeneration for the treatment of periodontitis (104). This not only improves the safety and efficacy of stem cell therapy without causing extra side effects but also provides a viable and promising approach to the clinical treatment of periodontitis.

## Conclusion

Over the past 30 y of exosome research history, a great deal of research and exploration has been conducted on the function of exosomes under different physiological and pathological conditions, and the knowledge has expanded very rapidly. For exosome isolation and identification, EVS, ENPs and most other secreted products can first be recovered by filtration or polymer-based precipitation global concentration methods. Next, EV subtypes are separated using differential centrifugation with varying centrifugal force/time depending on size and/or weight. Further separation of EV subtypes and ENPs can be achieved by bottom-up flotation into a density gradient (DG) or top-down DG or density pad separation. Finally, isolation of the most specific exosomes is achieved by immunoprecipitation using antibodies directed against specific surface proteins of EVS (105). Many studies have shown that exosomes themselves have signaling capabilities that allow for wireless communication between cells. On the one hand, exosomes can act as carriers of cellular waste, and on the other hand, they can be used to build novel targeted drug delivery systems that can act on targeted cells and change the “future” of the cells. It is clear that exosomes have a strong influence on the development of inflammatory immune diseases and tumor progression and metastasis, inducing effective pro- and antitumor immune responses. The effect of activating immunity mainly depends on antigen presentation by exosomes, while the effect of immune inhibition by exosomes mainly depends on their carried ligands, proteins, and miRNAs (106). Recent evidence suggests that tumor-derived exosomes could be responsible for mediating systemic immunosuppression that antagonizes anti-PD-1 checkpoint therapy. Endogenous tumor exosomal PD-L1 and tumor exosome-induced PD-L1 are the two most obvious mechanisms of exosomal resistance against tumor immunity (107). In addition, exosomes are now found to be emerging biomarkers in the diagnosis and treatment of many diseases, with promising applications in the field of immunotherapy. Zhou et al. (108) established a novel integrated dual-channel 3D microfluidic chip that can synthetically detect multiple types of exosomal biomarkers (surface proteins and miRNAs), greatly avoiding false-positive rates and increasing the accuracy of tumor diagnosis and staging monitoring to 100% (108).

However, to date, the actual physiological and pathological functions of exosomes have not been well established, and research on immune cell-derived exosomes as biomarkers or targeted therapies for oncological or autoimmune diseases is still at an early stage and needs to be further developed. We also find that the existing research on exosomes is mainly focused on miRNAs, including intercellular communication, diagnostic and therapeutic biomarkers, and re-editing of immune target cells (109–111). Immune cells, pancreatic islets, and many other cells involved in metabolic diseases secrete large amounts of miRNAs in the form of exosomes. miRNAs can act sequentially in a paracrine or autocrine manner or reach the circulation and act in an endocrine manner (112). Nevertheless, the loading information and transactivation mechanism of exosomal miRNAs, their source and destination, as well as their working principle remain unclear. In addition to miRNAs, there are few studies related to the comprehensive systematic analysis of other exosomal components (e.g., lipids, proteins, mRNAs, lncRNAs, circRNAs, and DNA) and their interactions (113–118), reinforcing the need to determine whether there is an immune system influence and its principal mechanisms. As exosomes are derived directly from cell membranes, retain the properties of intact cells, and have a variety of adhesion proteins on their surface, they have become potential carriers for gene therapy, and their nano size and flexibility allow them to cross major biological barriers such as the BBB (119). In contrast to liposomal preparations, exosomes are naturally occurring secretory membrane vesicles with low toxicity, which can be inferred from their prevalence in the body where they are well tolerated. In addition, the inherent homing ability of exosomes hints at their potential utility in drug delivery. For example, melanoma-derived exosomes preferentially enter the anterior lymph nodes, and this homing ability could be used for targeted drug delivery (120).

Exosomes are small nano-molecules directly derived from cell membranes and retain the properties of intact cells. Thus, the emerging engineered exosome platform technology for nano-drug delivery, which has been heavily invested on in recent years, can exploit the powerful advantages of exosomes in clinical immune-targeted therapies (121, 122), particularly for the development of therapeutic agents for the treatment of autoimmune diseases or the prevention of organ transplant rejection. Ultracentrifugation is currently the most popular method for exosome isolation, but this method suffers from low throughput and purity. Liu et al. (123) developed an ultrafast-isolation system (EXODUS) that applies double coupled harmonic oscillations into a dual nanoporous membrane filter system to remove small particles like nucleic acids and proteins while capturing bigger-sized exosomes, enhancing the speed, yield, and purity of exosome separation. Furthermore, MDimune developed an exosome-based drug delivery platform, called the BioDrone™ platform, which produces large quantities of exosomes (cell-derived vesicles (CDVs) from various cell sources by extrusion, enabling targeted delivery of specific targets, reducing drug doses and side effects, and maximizing drug efficacy (124). However, the development of engineered exosome technology also faces some fundamental limitations that need to be overcome, including the molecular composition and size of exosomes, the quantity and quality of production, the maximization of biological function applications, and the inevitable interactions with other biological systems. It is believed that in the near future, more in-depth progress can be made in the field of exosomes, which will eventually lead to breakthroughs in clinical applications for patient treatments.

## Author Contributions

XZ and DX contributed equally to the writing of the manuscript. TW conceived and designed the work. YS and RH participated in its coordination and modification. All authors contributed to the article and approved the submitted version.

## Funding

This work is supported by Liaoning Province Natural Science Foundation [2020-ZLLH-47]. This work is also funded by Liaoning Province Science and Technology Plan Project [2017225054], Liaoning Province Key Area Joint Open Fund [2019-KF-01-01], and the Tumor Mass Spectrometry Center Project [ZP202013].

## Conflict of Interest

The authors declare that the research was conducted in the absence of any commercial or financial relationships that could be construed as a potential conflict of interest.

## Publisher’s Note

All claims expressed in this article are solely those of the authors and do not necessarily represent those of their affiliated organizations, or those of the publisher, the editors and the reviewers. Any product that may be evaluated in this article, or claim that may be made by its manufacturer, is not guaranteed or endorsed by the publisher.

## Abbreviations

EV, extracellular vesicle; TAM, tumor-associated macrophage; DC, dendritic cell; SLE, systemic lupus erythematosus; MSC, mesenchymal stem cell; MC, mast cell; HCC, hepatocellular carcinoma; DM, diabetes mellitus; GEX, glioma-derived exosome; MDSC, myeloid-derived suppressor cell; HNC, head and neck cancer; RA, rheumatoid arthritis; LPS, lipopolysaccharide; MS, multiple sclerosis; CRPS, complex regional pain syndrome; PE, plasma exchange; GMSCs, gingival tissue-derived mesenchymal stem cells.
